# Economic Evidence on Cost Sharing and Alternative Insurance Designs to Address Moral and Behavioral Hazards in High-Income Health Care Systems: A Systematic Review

**DOI:** 10.3390/jmahp12040027

**Published:** 2024-11-14

**Authors:** Marlon Graf, James R. Baumgardner, Ulrich Neumann, Iris P. Brewer, Jacquelyn W. Chou, A. Mark Fendrick

**Affiliations:** 1 Precision AQ, Bethesda, MD 20814, USA; jim.baumgardner@precisionaq.com (J.R.B.); iris.brewer@precisionaq.com (I.P.B.); jacki.chou@precisionaq.com (J.W.C.); 2 Johnson & Johnson Innovative Medicine, Titusville, NJ 08560, USA; uneuman1@its.jnj.com; 3 Center for Value Based Insurance Design (V-BID), University of Michigan, Ann Arbor, MI 48109, USA; amfen@med.umich.edu

**Keywords:** moral hazard, behavioral hazard, value-based insurance, efficiency, patient out-of-pocket costs, patient cost-sharing, patient cost exposure

## Abstract

In health insurance, “moral hazard” describes the concept that coverage without an out-of-pocket cost to consumers could result in health care utilization beyond economically efficient levels. In response, payers in the United States (US) have designed pharmaceutical benefit plans with significant cost exposure (e.g., co-pays, co-insurance, or deductibles). While substantial evidence links patient cost exposure to reduced drug spending, it remains unclear to what degree this translates into greater efficiency or an indiscriminate drop in overall consumption also reducing needed utilization. We conducted a systematic literature review to understand whether commonly implemented utilization management (UM) strategies and insurance designs with a behavioral or value-based (BID/VBID) component have been explored as tools to mitigate moral hazard and to assess how cost-sharing policies and innovative insurance designs impact consumer spending. Eligible studies compared conventional cost-exposure policies to BID/VBID, including tiered cost-sharing and other UM strategies. We found that broad implementation of patient cost exposure is not well supported by empirical evidence assessing efficiency—defined as the use of clinically appropriate services with value at or above the marginal cost of health care utilization in the contemporary US setting. As a result, payers and policy makers alike ought to explore insurance alternatives that more closely align health care consumption incentives to value of care.

## 1. Introduction

In the context of health insurance, the term “moral hazard” is widely used for the economic intuition that insurance coverage can lead to an inefficient increase in health care use since out-of-pocket (OOP) costs to the consumer are decreased below the true cost of the service [1]. Beneficiary cost exposure (e.g., cost-sharing, co-pays, co-insurance, and deductibles) [2] has been promoted as a response to reduce moral hazard in health insurance, often referred to as “skin in the game.” A robust evidence base demonstrates that cost-sharing can be effective in reducing health care consumption and thereby spending on the health care services to which it is applied [1,3,4,5]. Relative to full insurance without cost-sharing, introducing cost-sharing can be viewed as a tax on health care consumption with relatively more cost burden placed on those who end up using medical services. Benefit design approaches with heightened consumer cost exposure have gained prominence in the US, as evidenced by the rapid growth in High Deductible Health Plans (HDHP) in the commercial health insurance market [6], which typically apply an OOP amount to all care, regardless of value (unless otherwise mandated). This has led to a growing cost burden in addition to premiums shouldered by US patients; reported to be USD 471.4 billion in direct OOP payments in 2022 [7]. Such estimates likely undercount the true magnitude experienced by patients and unpaid caregivers due to various health-related OOP expenses that are generally not accounted for in conventional measures [8,9]. Furthermore, blunt cost-sharing instruments that indiscriminately increase OOP costs of all care to consumers have the potential to decrease access to necessary care and worsen health disparities [9].

### 1.1. Research Background

In advancing benefit designs to counteract moral hazard, the predominant emphasis has been on curtailing aggregate medical consumption rather than ensuring expenditure is commensurate with the value of care delivered. However, the fact that beneficiaries respond to changes in price alone does not necessarily mean their consumption can be considered more efficient. Traditional economic theory posits that an efficient allocation of resources occurs when consumers or patients receive care with a marginal value that exceeds or equals its total marginal cost and forgo care with a marginal value less than its total marginal cost. Insurance can create consumption inefficiencies by reducing the OOP price to a point where the consumer or patient perceives a health care good to be priced below its total marginal cost [10]. The resulting consumption of care with a marginal value below its total marginal cost is an example of an ‘inefficient’ moral hazard. Conversely, research also suggests that individuals might not receive treatment that would be efficient to consume, even at a negligible or no OOP cost, for instance, due to a lack of actionable information about treatment benefits [11].

These conceptual concerns have been amplified by a growing body of empirical literature challenging the notion that increased beneficiary cost exposure, widely implemented as the primary remedy to moral hazard in contemporary benefit design, is in fact appropriate to address excess health care spending [12,13]. Recent studies suggest beneficiaries are equally sensitive to price changes for both high and low-value care, reducing consumption of both indiscriminately, without a clear ‘rational’ perspective on their health or resultant costs to the system [14]. For instance, a recent rapid literature review assessed the impact of cost-sharing reductions on the utilization of preventive care, finding lower cost-sharing was associated with increased use of preventive care in most cases, especially for individuals who were financially vulnerable [15]. Consistent with this rapid review, our review defines preventive care as guideline-recommended routine health care including screenings, check-ups and counseling.

While increased cost exposure reduces the immediate use of care, the resultant health care decision-making may be subject to various informational and behavioral biases, and the implications on downstream health care utilization and equity effects are likely substantial, yet not well-understood. A known challenge of financially empowering consumers in health care decision-making is their inability to ascertain OOP costs until after care is delivered in most clinical contexts [16,17]. Reliance on cost exposure across a broad range of services or products without differentiation of value may lead to an inefficient allocation of health care resources.

In view of these dynamics, the idea of a ‘myth of moral hazard’ in health care has gained prominence [18]. Some researchers have sought to re-define the term in the economic literature, referring mainly to the observed change in consumption in immediate health care services as a result of having insurance as opposed to attaching any implications on the morality of ‘overuse,’ value-based decision-making, or welfare optimization [1,4,19]. Others have noted that at least some part of the observed moral hazard effect is ‘efficient’ as long as it refers to the use of medically necessary services that are valued by the beneficiary but cannot be afforded by them due to financial constraints [20,21]. Simply put, patients might not adhere to treatment under increased cost exposure because they can no longer afford it, not because they do not value it. Lastly, it was argued that a focus on cost optimization directed primarily at the responsibility of the demand side (i.e., consumer of health care) overlooks the potential for ‘moral hazard’ on the supply side (e.g., the provider), as well as barriers to transparency and market failures in the system. Furthermore, influenced by the learnings from behavioral economics, recent studies have proposed the additional concept of ‘behavioral hazard’ to capture confounding non-financial factors that ought to be considered alongside monetary incentives when thinking about efficient benefit design [1,11,18,22].

In view of how the terminology has evolved in the existing literature, we define moral hazard as the demand for inefficient health care that occurs because people are insured and we define behavioral hazard as the phenomenon where insured consumers fail to use high-value care or inefficiently use low-value care because of psychological biases or a lack of information [11]. Both are understood as sources of health care misutilization that benefit designs seek to correct.

### 1.2. This Study’s Relevance for Health Care Decision-Making

Questions around the ability of traditional cost-exposure approaches to address both moral and behavioral hazards are not purely academic but have important real-world implications for insurance design. The substantial prevalence of US households—many of which are insured—facing medical debt indicates that affordability and unpredictability have become broader challenges of cost exposure for medical care beyond the uninsured [23,24,25]. A long-standing concern about cost exposure relates to health equity and the disproportionate burden it may pose on sicker, lower-income or historically marginalized populations [26,27]. For example, HDHPs were shown to disproportionally affect Black patients on several measures of medication access among cancer survivors [28], while reducing co-insurance was identified as a vital step in dismantling systemic health disparities [29,30,31,32].

Thus, insurance providers have in turn come under pressure to develop more nuanced, dynamic and equitable benefit designs [33]. As a result, value-based insurance designs (VBIDs) have emerged as potential solutions to encourage use of services when the health benefits exceed the costs and to discourage use when outcomes do not justify the cost [34]. These models may still incorporate elements of targeted patient cost exposure, but with more nuanced provisions to weigh both affordability and clinical value [34]. In the case of COVID-19 vaccines, for example, the US government fully covered both vaccines and testing as critical, high-value care [35]. Furthermore, a 2021 systematic review examined the utilization impact of the Patient Protection and Affordable Care Act (ACA) preventive care mandate, finding most published studies showed increases in the use of fully covered services following the elimination of cost sharing [15]. Importantly, studies that included socioeconomic status reported greater increases in utilization of preventive services in financially vulnerable patients as compared to those with higher incomes, suggesting that the policy reduced disparities in the delivery of preventive care.

However, while VBIDs are widely recognized among health policy researchers as an approach to improving the efficiency and value of health care spending [36], they are far from the dominant insurance design in the US. This is despite the fact that under the ACA, private health plans are obligated to provide coverage for a range of recommended preventive services, including evidence-based screening and counseling, routine immunizations as well as preventive services for women, and may not impose cost-sharing (such as co-payments, deductibles, or co-insurance) on patients receiving those services.

Given both the academic debate and real-world relevance, it is important to explore empirical research that has been conducted about contemporary insurance designs in the context of moral and behavioral hazards. Past studies have synthesized existing evidence regarding the impact of increased cost exposure on utilization [37]. However, most studies have not evaluated whether changes in utilization would be considered high value or low value, nor have they compared insurance designs to each other or assessed whether certain populations have disproportionally been affected.

## 2. Our Methodological Approach

Existing research offers few indicators on how to optimize benefit design to account for behavioral and moral hazards, often presenting conceptual arguments [11,12,22] or case studies without study controls [38,39]. To address this gap, we sought to investigate whether different cost-exposure designs have been studied with respect to efficient and inefficient moral hazard. We explored if and how commonly used utilization management (UM) strategies (e.g., prior authorization, step therapy, quantity management, drug utilization review, site of service steerage, and the formulary exception process) [40] as well as alternative insurance designs (e.g., zero-co-pay programs, VBID models) have been compared in the empirical literature with respect to their ability to curb inefficient moral hazard or otherwise promote efficient and equitable use of health care. The methods for this study as well as high-level findings were previously published in a poster at AMCP Nexus [41].

### 2.1. Systematic Literature Review

The literature search strings were carefully developed by PhD economists with domain expertise in the academic study of health economics and insurance design (authors MG, JRB), and then refined by experienced methodologists within the authors respective organizations. We then searched the literature for studies that compared the impacts of different cost exposure policies with each other or with commonly implemented UM or behavioral insurance designs (BIDs). We extracted key results related to utilization and efficiency. Given the importance of addressing health equity considerations, we also probed whether studies considered any distributional consequences.

Study eligibility criteria were defined in terms of the population, interventions, comparators, outcomes, and study design (PICOS) structure outlined in Table 1. Based on the eligibility criteria, we proceeded to identify relevant studies by searching the EconLit database via OVID and the MEDLINE database via PubMed from 1 January 2000 to 13 February 2023. The review timeframe was limited to studies published in 2000 or later for two primary reasons: First, our study sought to identify publications that present an evolved and more nuanced interpretation of moral hazard, whereby some increased consumption under insurance may be efficient, and morally hazardous consumption does not mean overconsumption of any health care good or service, but instead only refers to overconsumption of low value or inefficient care. Additionally, we incorporate the concept of behavioral hazard in this review, which describes the underconsumption of high-value or efficient care. The evolved interpretations of moral hazard as well as the concept of behavioral hazard were first introduced by a series of publications between 2003 and 2018 [11,14,42,43]. Second, our study aims to compare the efficiency outcomes of cost-exposure policies (such as co-pays and co-insurance) with alternative insurance mechanisms that are popular in the current US health care system. Many of these alternative policies such as VBIDs (first conceptualized in 2001) [44] and HDHPs (formally instituted in 2003) [45] were not around prior to 2000, meaning the insurance (and health care) landscape differed strongly and comparisons with any alternative insurance design pre-2000 would not be an accurate representation of the tools currently at the disposal of US-based payer organizations.

Studies identified from each source were merged and duplicate records were removed, and the respective search strategies are presented in Table 2.

As outlined in Table 3, studies were selected if they (1) evaluated existing or conceptual cost exposure policies in health and included a comparison of policies focused on beneficiary cost exposure through cost sharing, utilization management, or other alternative insurance design, and (2) applied a rigorous quantitative, qualitative, or mixed methods design with either theoretical or empirical approaches of moderate or high quality, as determined by Dixon-Wood et al.‘s quality assessment prompts [46]. We followed Rostamkalaee et al. [47], who also used the Dixon-Wood et al. criteria, which are specifically targeted toward the systematic quality appraisal of a complex, cross-disciplinary and diverse body of evidence related to the study of policies impacting health care access and outcomes.

Two graduate-level trained literature domain experts each independently reviewed a sample of 30 abstracts identified by the search according to the selection criteria, with the exception of the outcome criteria, which were only applied during the screening of full-text publications. This sample of 30 screened abstracts was then reviewed separately by three other health economics researchers to ensure screening results were in alignment with overarching study objectives. Following the sample screening review, the entire team of authors met and discussed the overall screening process. Once this adaptive screening process was concluded and the research team had aligned on the screening criteria and practice, the two primary reviewers continued to screen the remaining abstracts according to the selection criteria specified above and the takeaways from the sample screening process. Articles were excluded if they did not directly compare at least two different types of insurance designs.

**Figure 1:**
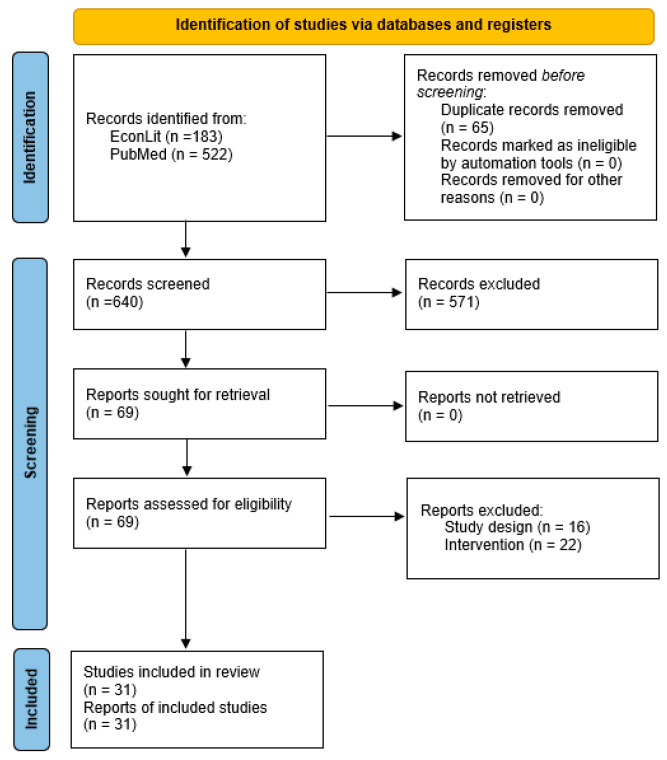
Literature review flowchart (PRISMA).

All studies identified as eligible during abstract screening were then screened independently at a full-text stage for purposes of inclusion in the final extraction by the same two reviewers. Any differences in determinations made by the two reviewers were reconciled by the additional authors. Full-text studies identified at this stage were included for data extraction. The process of study identification and selection is summarized with a Preferred Reporting Items for Systematic Reviews and Meta-Analyses (PRISMA) flow diagram in Figure 1 [48]. This study was conducted according to general systematic evidence review practices pertinent to this research domain and reported according to the PRISMA checklist [49]; the review was also registered in Open Science Framework (registration number: osf.io/wm5f2).

### 2.2. Data Extraction and Analysis

One reviewer extracted relevant data from the final list of included studies into a pre-specified extraction worksheet, developed by the research team after reviewing the final extraction sample. Data were stored and managed in PRESTO, Precision AQ’s internal literature management software tool, and then exported into a Microsoft Excel workbook for analysis. Specifically, the team extracted general information on the study (authors, title, year, citation, study location, etc.), study design and data sources used (e.g., retrospective analysis, economic experiment, simulation study, etc.), involvement of pharmaceutical products or health services, disease areas covered and study populations included, treatment and definition of the concepts (i) moral hazard, (ii) behavioral hazard or (iii) efficiency/value, insurance designs evaluated (e.g., type of cost-exposure design and/or traditional UM design), and study findings and conclusions (comparative value, differences in health, economic or equity outcomes between compared insurance design alternatives). Additionally, following the approach in a systematic review of similar literature by Rostamkalaee et al. [47] study quality was evaluated according to Dixon-Wood et al.’s checklist specifically developed for the systematic quality appraisal of a complex, cross-disciplinary body of evidence related to policies of health care access and outcomes [46]. All included studies were determined by the research team to be of medium or high study quality.

## 3. Results

### 3.1. Study Sample

Initial searches in PubMed and EconLit yielded 705 unique abstracts, 70 of which were flagged for full-text screening and 31 of which were included in the final extraction sample for this study. The PRISMA diagram in Figure 1 summarizes the process of study identification and selection. The final list of studies (Table 4) included 19 retrospective data analyses, six natural experiments, three economic models, two difference models and one computer simulation, of which, 22 studies were conducted in the United States, five in Canada, two in the Netherlands, and one each in Spain and Germany. Six studies covered the concept of behavioral hazard, framed primarily as the underuse of essential or highly efficient care [50,51,52,53,54,55] and two studies explicitly compared a beneficiary-facing cost-exposure policy (co-pay, cost-sharing) to a UM strategy (prior authorization) [56,57].

Eighteen papers discussed impacts on pharmaceuticals [50,52,53,55,56,57,59,60,64,66,67,68,69,70,71,74,77,78] and 13 papers discussed impacts on health service utilization [51,54,58,61,62,63,65,72,73,75,76,79,80]. Some papers were focused on health care utilization among the general population [54,55,59,60,61,65,69,70,71,72,75,79], while other studies focused on cardiovascular disease [57,66,68,77,78], chronic unspecified disease [53,56,73,74], and diabetes [50,64,67], as well as mental health [52,62], cancer [76], colonoscopy [58,80], low-value services [63], and preventive services [51].

### 3.2. Definitions of Policies Emerging from This Review

The literature searches in this systematic review were focused on studies comparing insurance policies that included cost exposure for beneficiaries to other policies commonly used by insurance companies. Specifically, we defined cost exposure as any approach that passes along some portion of the cost of services to the insured individual as an OOP expense. This exposure could happen through three mechanisms: (1) deductibles, where beneficiaries must meet a minimum OOP spending threshold before the insurer begins to pay; (2) co-insurance, where beneficiaries are required to pay a percentage of costs and finally, (3) co-pays, where beneficiaries are required to pay a fixed amount per service received.

Based on the review of the studies we evaluated, these cost-exposure policies were compared to either another cost-exposure policy or to other policies such as (1) consumer rebates providing money back to beneficiaries who stay below a predefined threshold of health care utilization; (2) tiers placing drugs or health services into different degrees of coverage based on criteria determined by the insurer; (3) VBIDs making coverage and reimbursement decisions based on the perceived value of a good or service; (4) prior authorization, which requires pre-approval from the insurer for prescriptions to qualify for coverage; (5) step therapy, which requires use of lower cost therapies for a given condition before “stepping up” to more expensive therapies and, finally; (6) reference pricing, which determines reimbursement rates based on reference points or benchmarks. While benefit designs often include a deductible along with another cost-exposure component, we focused on the components that differed among the plans that were compared in a given study.

### 3.3. Understanding of Moral and Behavioral Hazard

Among the studies included in this systematic review, 11 papers directly covered the topic of moral hazard in health care [52,55,60,61,62,63,67,73,75,79,80], six papers directly referenced the concept of behavioral hazard in health care [50,51,52,53,54,55] and two papers covered both concepts together [52,55].

Moral hazard across most studies was defined as overconsumption of health care goods or services, driven by generous insurance coverage and resulting low cost burden to consumers [52,60,62,67,73,75,79]. Only three studies added an efficiency dimension to moral hazard, whereby moral hazard was defined as the overconsumption of low-value care, rather than just the overconsumption of care in general [55,63,80]. Lastly, while most studies framed moral hazard as a consumer-side issue, one study explicitly focused on provider-side moral hazard, finding that a higher diagnostic coding intensity in US insurance claims is driven by the fact that US health care providers diagnose and treat more conditions due to “generous fees, frequent patient contacts, and abundant test results” [61].

Behavioral hazard was described consistently as an underuse of high-value care, driven by excess cost-exposure [50,51,52,53,54,55], and, in studies addressing both concepts together, was framed as the opposite of moral hazard: one study suggested cost-sharing for low-value services ought to be increased to curb moral hazard, while it should be reduced for high-value services to combat behavioral hazard [55]; similarly, another study cautioned that cost-sharing policies could have two important unintended consequences: increased utilization of other non-drug health services that may also be inefficient or low value, as well as reductions in essential drug use [52].

### 3.4. Cost-Exposure Consequences: Affordability, Outcomes, Health Care Efficiency and Equity

This systematic literature review largely yielded studies comparing the consumption effects of health insurance policies. However, it is also important to consider any other impacts beyond the utilization of high- and low-value health care.

From a payer perspective, the overall cost and utilization of health care goods and services are usually considered a primary criterion for coverage and reimbursement decisions. Thus, a policy that is beneficial in the view of payer organizations would lower aggregate health spending and utilization. Ideally, any reductions in utilization would focus on low-value care, but most studies simply measure the overall consumption of the given treatment without considering the value.

Across the studies reviewed here, the impact on utilization and spending was recorded frequently, though measured somewhat inconsistently. Specifically, 27 of 31 papers examined how changes in cost exposure impacted utilization [50,51,52,53,55,56,57,58,59,61,62,63,64,65,66,67,68,69,71,72,74,75,76,77,78,79,80], with findings largely consistent with the results of another recently completed systematic review, which indicates that as patient costs increase, health care utilization decreases [37]. Furthermore, policies that implemented differential cost-exposures (e.g., through a VBID or tiered design) based on perceived value tended to see greater reductions in the utilization of low-value care [55,58,66,68,69]. Deductibles appeared to be more effective in steering health consumption than consumer rebates across multiple studies [54,62,75,79], while implementing a VBID led to improved access and adherence to high-value medication and hence, improved health outcomes, compared to policies with greater patient cost-exposure, which did not [50,55,59,63,64,74,77,78].

Additionally, from a societal perspective, both aggregate health care expenditures and broader welfare effects should be considered critical components of novel insurance policies. However, while most of the studies included in this review accounted for utilization impacts of the policies under comparison, broader societal impacts were only covered by a fraction of studies and results were not reported in a consistent or comparable fashion: Specifically, one study stated that consumption of low-value care was a key driver of U.S. health care expenditures [55], and another study suggested that low-value care was more prevalent in settings with low patient cost-exposure [65], but only 7 of 31 papers explored outcomes related to the value of care (e.g., high vs. low value, efficient vs. inefficient) [53,55,58,63,65,73,75]. Examples of low-value care included sleep studies, advanced imaging services, endoscopies, and surgeries, when used widely and without established medical need [63], and studies found consumption for these services to be more sensitive to price changes than consumption of pharmaceuticals [53,73,75].

Additionally, nine of 31 papers explored the differential impact of cost-share/alternatives for different sub-populations, including those of high/low socio-economic status (SES), individuals with chronic illnesses, as well as the elderly [54,59,60,62,74,75,77,78,79]. Several of these studies suggested lower-income consumers were more likely to adjust their health care consumption in response to a change in deductibles than in response to consumer rebates or refunds [54,75]. Additionally, increases in cost-exposure were associated with reductions in medication adherence for lower-income patients [59,77,78], while VBIDs were found to mitigate some of these effects [74].

### 3.5. Optimizing Benefit Design: Comparative Assessment of Insurance Policies

Detailed comparisons between cost exposure and so-called control designs are summarized in Table A1. Among the studies identified, the most common comparisons were between different applications of cost-exposure policies (e.g., co-pay, co-insurance and deductible).

The largest comparison pairing was between co-pay and co-insurance, with 13 studies. Some studies found that beneficiaries generally tended to reduce health care spending similarly under both co-pay and co-insurance policies [60,62,65,67,70,73], while others showed mixed effects on compliance and medication adherence [50,59,64,77,78]. Two studies showed that, relative to co-insurance, co-pays did not increase the utilization of health care services [52,73] while another showed a smaller impact on preventive service utilization [51].

Among the four studies comparing co-pays to deductibles, no clear trend emerged. In some cases, deductibles were associated with increased use of lower-cost drugs [53], while co-pays per each day of a hospital stay might be associated with declines in inpatient hospital admissions and days [72]. One study compared co-pay to a combination of deductible and co-insurance in asthma patients, concluding that while both options reduced monthly spending on inhalers, the effect was larger for the combined deductible and co-insurance plan [60]. Lastly, one study compared co-pays to deductibles, concluding that co-pays have a less positive and sometimes negative impact on household welfare [79]. One study included both deductible and co-insurance, finding that the plan with more comprehensive coverage (higher premium, lower co-pay, lower co-insurance, lower deductible) resulted in higher total health care costs and higher consumption of health care visits which was deemed an inefficient use of health care resources [73].

The next set of comparisons was between tiering policies and cost-exposure policies. For the purposes of this review, tiering was defined as any insurance policy that applied differential pricing or access to treatments or services. Three studies compared tiering to co-pay, finding that in general, introducing tiers led to decreases in total spending on pharmaceuticals, especially for those on higher tiers, as well as a relative increase in both generic and preferred treatment options [66,69,76]. Two papers assessing the differential effects of tiers and co-insurance suggested that implementing tiered coverage policies leads to small reductions in overall health care utilization and spending [68] as well as small increases in generic fill rates [71]. Finally, one paper comparing tiers to deductibles concluded that tiered plans led to lower average spending than deductible plans, at a lower OOP cost to consumers [58].

Three studies compared cost-exposure policies to VBIDs. However, it must be stated that in order to be classified as VBID in this review, a study had to explicitly refer to one of the insurance options as a “value-based insurance design”. Other policies restricting coverage for specific therapies (such as tiers, prior authorization or step edits) may do so based on value or efficiency, but if not explicitly referred to as a VBID, would have been categorized under a category other than VBID in our review. One study compared a VBID design to co-pay and co-insurance, respectively, stating that compared to non-VBID co-pay, VBID in the form of co-pay programs led to increased life expectancy gain attributable to health care and varying effects on overall health expenditures [55] and that compared to co-insurance, VBID in the form of co-pays significantly reduced the use of targeted low-value services and substantially increased cost-sharing for targeted services, but did not lead to overall cost savings [63]. When comparing VBID to a deductible plan with no VBID, patients overall maintained adherence to chronic disease treatments, while those in a subset of higher income neighborhoods with a no VBID plan had reductions in adherence while those with a VBID plan maintained a consistent level of adherence [74].

Lastly, four studies compared cost-exposure policies to other policies such as consumer rebates, prior authorization, and step therapy, arriving at the conclusion that deductibles were more effective than consumer rebates at curbing overall health care expenditures [55,75] and that low cost-sharing for generics, large differentials in cost-sharing for generic versus brand drugs, and tools such as prior authorization and step therapy were all associated with higher generic drug use [56,57].

One study compared reference pricing, structured as a reimbursement limit for a colonoscopy, to real-world cost-sharing, concluding that reference pricing increases demand for low-priced providers and leads to a sizable reduction in the average cost per colonoscopy [80]. Another study concluded that narrow panel health plans do better at reducing costs and utilization than high cost-sharing plans, relative to preferred provider organizations [61].

## 4. Discussion

Two recent systematic reviews surveyed the literature on both cost-sharing and moral hazard. Fusco et al. [37] sought to understand the current evidence base regarding cost-sharing and four key outcomes: medication adherence, clinical outcomes, health care resource utilization, and health care cost. Their review identified that higher cost-sharing led to reduced medication adherence in 63 studies (84% of studies), and the authors caution that cost-sharing may potentially result in increased inpatient care and decreased outpatient care. The authors concluded that cost-sharing may be too blunt to achieve optimal outcomes, and likely reduces all care, not just unnecessary care. Like Fusco et al., we were also interested in understanding the impact of cost-sharing on similar outcomes.

Building upon the previous review, we aimed to delve further into the type of care impacted and to draw comparisons between specific cost-sharing strategies in relation to the economic theory of moral hazard. Our findings are consistent with Fusco et al.: evidence linking cost sharing to the efficient use of health care remains sparse. Furthermore, our comparative analyses are inconclusive regarding which benefit design configuration would be ideal to achieve efficient health care use. Though Fusco et al. [37] reviewed the landscape on outcomes, they did not address the moral hazard rationale for cost-sharing in their review.

On the other hand, a systematic review by Rostamkalaee et al. [47] centered on studies related to moral hazard. Unlike our study, Rostamkalaee et al. [47] did not evaluate the outcomes of moral hazard benefit designs but instead summarized and cataloged available benefit tools based on the type of behavior change each tool is intended to achieve. Our study focused specifically on comparing cost-sharing benefit designs with other insurance designs, including various forms of cost-sharing and utilization management strategies, allowing us to provide a more detailed analysis of the impacts across different cost-sharing approaches.

**Figure 2:**
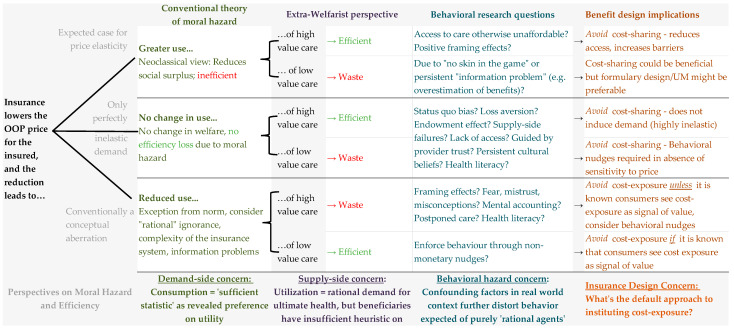
Decomposition of moral hazard relative to benefit design implications: evolved considerations of efficiency from conventional theory [10,81,82] to extra-welfarist perspectives [83,84] and behavioral research question [11,85].

Figure 2 synthesizes the economic concepts studied in this review, illustrating that the health and societal outcomes associated with the observed excess consumption under insurance are inadequately understood by conventional moral hazard theory.

The normative implications from the neoclassical moral hazard model [81,82] have been debated in the conceptual economic literature for several decades [83]. Our review finds that a growing body of empirical studies adds to those theoretical challenges by revealing real-world behavioral confounders, inherent limitations of patient information landscapes, and emergent distributional issues. At the core, we find that few studies address efficiency by properly categorizing the value of care impacted. Instead, most studies simply measure any impact on care/utilization without determining whether that impact would be considered desired or efficient. While we can still reaffirm wide support for the neoclassical view that demand is responsive to price for a substantial segment of health plan expenditures [84], conclusions about the welfare implications of those changes in consumption require more nuanced studies.

For research involving pharmaceuticals, adherence is the most studied intermediate endpoint, and our findings are consistent with a body of literature showing a consistent impact of cost exposure on drug initiation and continuation—the greater the cost-sharing, the worse patient adherence [86,87,88,89]. This systematic review could not identify evidence that documented the ultimate consequences of an insurance policy such as cost-exposure on patient outcomes in a reliable, comparative design. Consistent with the objective of evidence-based policy making, such research should arguably precede the adoption of insurance designs that shift historical amounts of cost to patients, in particular since it is understood from several individual studies that cost-related non-adherence is correlated with disease complications, adverse outcomes such as hospitalizations, mortality and overall health care cost increases [88,90,91,92,93,94,95]. The implication of even small changes in cost exposure needs to be properly investigated—for example, a recent study was able to link a small increase in prescription OOP to a substantial increase in deaths in Medicare [96].

In the studies included in this review, VBID models stand out as ways to first identify the value of care on the ‘supply side’ and then implement cost-sharing designed to deter the use of low-value or non-essential care and promote efficient care. Studies on VBID models suggest that finding effective ways of deterring low-value care use would more successfully control for moral and behavioral hazards than uniform patient cost exposure.

Based on the studies we reviewed, it remains unclear whether increasing the cost to patients is any more suitable in managing moral hazard than mechanisms to drive compliance with clinically driven formulary management. Considering heterogeneous therapeutic effects in different populations, a preference for clinical nuance is hard to reconcile with an approach that imposes uniform cost (or a uniform co-insurance rate) on patients for all treatments in a class or all treatments in a formulary tier, e.g., specialty drugs. It should be noted that price elasticities of demand were found to vary considerably across disease areas and medication classes, which suggests that differential cost-sharing could improve economic efficiency. The optimality of uniform cost-sharing becomes even more tenuous in light of studies that suggest these elasticities may be a result of imperfect information or other factors that result in behavioral hazards. These dynamics may result in inefficient use of health care resources not just for the individual patient, but at the health system level, creating misalignment and misallocation of resources.

Furthermore, it should be noted that the large volume of the demand for medical treatments, and the cost-sharing associated with their use by patients follows a process of physician attestations and insurance or PBM adjudication through prior authorization, even for chronic disease patients with established medical needs or acutely ill cancer patients. It is widely accepted that there is no moral hazard-driven consumption in these situations, yet cost-sharing obligations often remain unadjusted [12,97]. Particularly illustrative examples of customary co-insurance obligations without moral hazard rationale can be observed in the context of insulin use or emergency care for accident victims who are at risk of bleeding to death. These situations inherently lack any incentive for overuse, as patients have no control over the timing or necessity of care; they require medical attention to receive life-saving treatment deemed medically essential. If true in extremis, the obviousness of these examples raises questions about how they have informed the view on moral hazard more broadly, such as in many other areas where considerable payer utilization management routinely precedes prescribed therapy use.

Put pointedly, including a low-cost essential medicine in the pre-deductible phase can be as value-driven as eliminating all cost-sharing for a high-cost gene therapy prescribed to a rare disease patient based on a biomarker. Far from being theoretic explorations of moral hazard theory, the advent of precision medicine brings these considerations into the realm of imminent choices for value-based formulary design.

In summary, our review finds that recent empirical studies offer scant contemporary evidence that beneficiary “skin-in-the-game” should be used as the default mechanism to reduce waste or enhance efficiencies in health care consumption. Indeed, when the efficiency of the consumption change was measured, “skin-in-the-game” policies often resulted in an inefficient outcome. Instead, the efficiency was more likely not assessed at all, with the focus solely on overall consumption levels, regardless of efficiency.

Our review also suggests that neglecting behavioral factors in health insurance modeling can lead to considerable misinterpretations of consumer decision-making with extensive welfare implications. Future research should explore the sustainability of extrinsic rewards on inherent motivation towards personal well-being. Due to the presence of behavioral hazards, merely adjusting financial exposure will likely be insufficient, and non-monetary instruments should be explored as a complementary strategy beyond benefit design to incentivize behavior. In addition, the present study focuses primarily on cost-exposure policy in high-income countries and expanding the study setting to include evidence from low and middle-income countries may provide different insights regarding responses to financial and behavioral incentives that are not currently captured.

### Strengths and Limitations

The strict approach required for study identification in SLRs is both a strength, as it ensures comparability of the evidence base and a limitation. For example, an SLR of clinical trials greatly benefits from high levels of rigidity, while SLRs in a space that is less well defined and in which terminology is not consistent might suffer from a lack of flexibility. To ensure that we were creating a comprehensive and representative dataset, while also consistently applying definitions and screening criteria, the team took an adaptive approach, screening papers in small groups and ensuring consensus was reached by the research team on inclusion and exclusion. This adaptive approach combined a strength of a targeted literature approach, which allows learning from studies reviewed early in a search and applying those learnings to identify additional relevant papers, with the strength of an SLR, where a structured framework for inclusion/exclusion allows for the identification of studies that can be assessed and constructed into a data source.

Additionally, generalizability and applicability of our findings are in part subject to how quickly the evidence may have changed since we conducted our search. As such, our review may have missed studies that were published at the time of our search date if they had not yet been indexed by EBSCO and Medline. There is also a risk of publication bias as some studies fail to be published and others are published only in abstract form, which presents limited information. However, our search produced a number of studies that were inconclusive regarding the comparative finding between insurance designs, suggesting that publication bias may play a smaller role in this particular topic.

While we recognize that our selected articles were all published in the English language, which may present a limitation, no articles were excluded solely on the basis of not being published in English as part of our search design. Additionally, articles published in other databases and not in EconLit or PubMed may not have been included in this search.

Furthermore, within the context of this SLR, studies were only categorized as VBID if explicitly referred to as such by the authors. Thus, while additional studies included in the review might determine cost exposure based on a value-based design or formula, they might have been attributed to a different category in our analysis. This means the amount of VBIDs discussed in the final included set of papers might be understated.

Lastly, due to the study inclusion criteria, articles that may have had cost-sharing arguments but did not compare a particular type of cost exposure to either a different type of cost exposure or to other insurance approaches were not included in the final set of papers. We believe such a conservative approach is grounded in the essence of furthering evidence-based policymaking where a sufficient amount of comparative evidence should have accumulated that encourages the adoption of moral hazard-backed policies, especially those with such widespread implications for millions of Americans.

## 5. Conclusions and Future Research

The broad-based imposition of demand-side (patient) cost exposure is not well supported by empirical evidence on “skin in the game” regarding efficiency—defined as the use of services with a value greater than or equal to their marginal cost—of health care utilization in the contemporary US setting.

If effective incentives are meant to target those with the most relevant insight and the ability to act on it, current US beneficiaries may not be as empowered as the advocates of consumer-driven health plans have long claimed.

Rising expectations to address health inequities and the distributional impact of OOP cost burdens appear to reach beyond traditional economic paradigms and actuarial models, but they merit researchers’ consideration in determining access to modern health technologies through commercial insurance [98]. Based on this review of the evidence, we thus conclude that more empirical research on cost exposure and insurance benefit design is sorely needed. We propose an updated conceptual framework to guide this work.

First, it is fundamental to delineate and characterize the high and low value of interventions tied to the imposition of cost-sharing. This entails considering not only the comprehensive and long-term health benefits associated with various care episodes but also integrating patient preferences regarding financial responsibilities, their health, and quality of life.

Second, research evaluation must transcend simplistic measures of demand to accurately gauge successful outcomes. The focus should be directed towards documenting individual clinical outcomes that signify enhancements in health and quality of life, in addition to the downstream economic impacts for the system and society. Such assessments must also consider the distributive effects and the implications for health equity tied to cost-sharing policies, thereby facilitating the recommendation of exact adjustments where necessary.

Third, it is imperative to augment the array of tools at the disposal of insurers beyond conventional mechanisms of co-payments, co-insurance, deductibles and formulary tiers where, e.g., every treatment in the specialty category has the same co-insurance rate. Insights from behavioral economics reveal a spectrum of additional approaches deserving of exploration. This includes examining the unique effects of removing negative financial incentives, such as deductibles, versus introducing positive incentives, like consumer rebates. Furthermore, strategies should also encompass the deliberate integration of “nudges” and the design of consumer choice architecture to guide beneficiaries’ decision-making where monetary incentives cannot succeed due to behavioral hazards.

Fourth, it is indispensable for moral hazard research on demand-side cost exposure to be melded with scrutiny of the economic incentives and goals on the supply side in the current health care economy. This includes physicians’ preferences and their financial interests, as well as incentives for providers of medical technologies. Research must examine the objective functions of insurance providers within current US plan designs which involve resource allocation and profit maximization. It remains an open question to what extent shifting costs to patients could mainly be an expression of rent-seeking behavior in violation of the original insurance contracts, as some have suggested [99,100,101]. What is evident is that without a critical broader perspective, patient cost-sharing strategies and any desired effects on transparency, enhancing competition, influencing prices, optimizing efficient resource utilization, and fostering broader adoption of therapeutic innovations will remain empirically unsubstantiated.

By addressing these four areas, research can provide a solid foundation for designing insurance benefits that are more responsive to the complexities of today’s health care economics, credibly leading to a more effective, equitable, and patient-centered health care system.

## Figures and Tables

**Table 1:** SLR eligibility (PICOS) criteria.

Criteria	Description
Population	Any—no restriction
Interventions	Consumers who were exposed to an insurance design that incorporated some element of OOP costs to the consumer (e.g., co-pay, co-insurance, high deductibles)
Comparators	Consumers who were exposed to an alternate insurance design that incorporated OOP costs
Outcomes	Any health care utilization outcomes Any economic or financial outcomes
Study design *	Economic theory paper Economic model Retrospective database analyses Systematic literature reviews Non-systematic literature reviews
Time	Only studies published from 1 January 2000 to 13 February 2023 were included

* Note: Our research questions are interdisciplinary in nature and, therefore, require a mix of study designs. Indeed, studies assessing the efficiency impact of cost-exposure policies could come from a variety of disciplines (public health, public policy, health economics) and could draw on a wide range of methods (program/pilot evaluation, natural experiment). Moreover, we did not want to exclude any conceptual studies that discuss the theory of moral or behavioral hazard in health care, without first verifying that said studies have some form of empirical validation.

**Table 2:** Literature search terms.

**EconLit Search Strategy (1 January 2000 to 13 February 2023, Search executed: 13 February 2023)**		
**Number**	**Strings**	**Hits**
1	(copay* or “co-pay*” or coinsurance or co-insurance or deductible or “high deductible health plan” or “high-deductible health plan” or “cost-sharing” or “cost sharing” or “out of pocket” or “out-of-pocket”).mp.	3035
2	(outcome or spending or low-value or “low value” or efficiency or inefficiency or utilization or expenditure or waste* or affordability or elasticity or elasticities or inexpensive or expensive or “high value” or high-value or elasticities or consumption).mp.	273,712
3	((insurance and design) or value-based or “value based” or “prior authorization” or “step edit” or “step therapy” or “formulary” or “utilization management” or “service steerage” or “quantity management” or “utilization review” or ((copay* or co-pay) and (coinsurance or co-insurance or deductible or cost-sharing or “cost sharing”)) or ((coinsurance or co-insurance) and (deductible or cost-sharing or “cost sharing”)) or (deductible and (cost-sharing or “cost sharing”))).mp.	5026
4	1 and 2 and 3 and (health*).mp.	212
5	limit 4 to yr = “2000–Current”	204
**PubMed Search Strategy (1 January 2000 to 13 February 2023, Search executed: 13 February 2023)**		
**Number**	**Strings**	**Hits**
1	(copay*[Title/Abstract] or “co-pay*”[Title/Abstract] or coinsurance[Title/Abstract] or co-insurance[Title/Abstract] or deductible[Title/Abstract] or “cost-sharing”[Title/Abstract] or “cost sharing”[Title/Abstract] or “out of pocket”[Title/Abstract] or “out-of-pocket”[Title/Abstract]) AND (outcome[Title/Abstract] or spending[Title/Abstract] or low-value[Title/Abstract] or “low value”[Title/Abstract] or efficiency[Title/Abstract] or inefficiency[Title/Abstract] or utilization[Title/Abstract] or expenditure[Title/Abstract] or waste*[Title/Abstract] or affordability[Title/Abstract] or elasticity[Title/Abstract] or elasticities[Title/Abstract] or inexpensive[Title/Abstract] or expensive[Title/Abstract] or “high value”[Title/Abstract] or high-value[Title/Abstract] or consumption[Title/Abstract]) AND ((insurance[Title/Abstract] and design[Title/Abstract]) or value-based[Title/Abstract] or “value based”[Title/Abstract] or “prior authorization”[Title/Abstract] or “service steerage”[Title/Abstract] or “step edit”[Title/Abstract] or “step therapy”[Title/Abstract] or “formulary”[Title/Abstract] or “quantity management”[Title/Abstract] or “utilization review”[Title/Abstract] or ((copay*[Title/Abstract] or co-pay[Title/Abstract]) and (coinsurance[Title/Abstract] or co-insurance[Title/Abstract] or deductible[Title/Abstract] or cost-sharing[Title/Abstract] or “cost sharing”[Title/Abstract])) or ((coinsurance[Title/Abstract] or co-insurance[Title/Abstract]) and (deductible[Title/Abstract] or cost-sharing[Title/Abstract] or “cost sharing”[Title/Abstract])) or (deductible[Title/Abstract] and (cost-sharing[Title/Abstract] or “cost sharing”[Title/Abstract]))) AND (2000:2023[pdat])	525

**Table 3:** Criteria for study selection.

**Study Inclusion**
Focus on Health Cost Exposure Policies The study must address either existing (i.e., implemented) or conceptual (i.e., modeled) policies that influence cost exposure within the health care sector. It must incorporate a comparison of policies aimed at modifying beneficiary cost exposure, namely: ○ Cost-sharing arrangements (co-payments, co-insurance or deductibles) with ○ Utilization management techniques (prior authorization, step therapy, quantity management, drug utilization review, site of service steerage, or the formulary exception process), or ○ Alternative insurance models or designs (e.g., VBID)
Scientific Study Design Eligible studies must employ a sound methodological approach recognized in the scientific study of health care access and outcomes questions, either: ○ Quantitative (e.g., cohort studies, case–control studies, randomized controlled trials (RCTs), retrospective database analyses, natural experiments, econometric and economic models, difference-in-differences studies, computer simulations) ○ Qualitative (e.g., case studies, focus groups) ○ Mixed methods approaches (e.g., explanatory sequential designs, exploratory sequential designs, convergent parallel designs)
Methodological Rigor: The study must be published in domain-specific peer-reviewed academic or scientific journals (as captured in the EconLit and Medline databases). Studies must meet established standards of methodological rigor, as moderate or high quality as assessed by applying Dixon-Woods et al. (2007)’s quality appraisal prompts [46]
**Study Exclusion**
Studies not meeting the inclusion criteria or lacking sufficient methodological approach to undergo quality appraisal.

**Table 4:** List of included studies.

Author Year	Title	Study Design	Location	Disease Area	Quality Assessment
Ackley 2022 [58]	Tiered cost-sharing and health care demand	Retrospective database analyses	United States	Colonoscopy and upper gastrointestinal endoscopy episodes	High
Aznar-Lou 2018 [59]	Effect of co-payment policies on initial medication non-adherence according to income: a population-based study	Natural experiment	Spain	General population	High
Braithwaite 2010 [55]	Can broader diffusion of value-based insurance design increase benefits from US health care without increasing costs? Evidence from a computer simulation model	Computer simulation	United States	Not specified	High
Dor 2004 [50]	Does Cost Sharing Affect Compliance? The Case of Prescription Drugs	Economic model	United States	Diabetes	High
Dormuth 2009 [60]	Effects of prescription co-insurance and income-based deductibles on net health plan spending for older users of inhaled medications	Retrospective database analyses	Canada	General population	High
Ellis 2016 [61]	Health Plan Type Variations in Spells of Health Care Treatment	Retrospective database analyses	United States	General population	High
Friedman 2019 [62]	The Effects of Three Kinds of Insurance Benefit Design Features on Specialty Mental Health Care Use in Managed Care	Differences in differences model	United States	Mental health	High
Gruber 2020 [63]	The effect of increased cost-sharing on low-value service use	Differences in differences model	United States	Low-value services	High
Hayen 2021 [54]	Does the framing of patient cost-sharing incentives matter? the effects of deductibles vs. no-claim refunds	Retrospective database analyses	Netherlands	General population	High
Henk 2018 [64]	Novel Type 2 Diabetes Medication Access and Effect of Patient Cost Sharing	Retrospective database analyses	United States	Type 2 Diabetes	High
Hoadley 2012 [57]	In Medicare Part D plans, low or zero co-pays and other features to encourage the use of generic statins work, could save billions	Retrospective database analyses	United States	Statin users	High
Huang 2022 [65]	Analysis of Affordable Health Care	Retrospective database analyses	United States	General population	High
Huckfeldt 2015 [53]	Patient Responses to Incentives in Consumer-directed Health Plans: Evidence from Pharmaceuticals	Retrospective database analyses	United States	Chronic disease (high cholesterol, hypertension, Type II Diabetes)	Medium
Kamal-Bahl 2004 [66]	How do incentive-based formularies influence drug selection and spending for hypertension?	Retrospective database analyses	United States	Hypertension	High
Karter 2007 [67]	Effect of cost-sharing changes on self-monitoring of blood glucose	Retrospective database analyses	United States	Diabetes	High
Klepser 2007 [68]	Effect on drug utilization and expenditures of a cost-share change from co-payment to co-insurance	Retrospective database analyses	United States	Hypertension	High
Landon 2007 [69]	Incentive formularies and changes in prescription drug spending	Retrospective database analyses	United States	General population	High
Li 2023 [70]	Generic Price Regulation and Drug Expenditures: Evidence from Canada	Retrospective database analyses	Canada	General population	High
Mager 2007 [71]	Relationship between generic and preferred-brand prescription co-payment differentials and generic fill rate	Retrospective database analyses	United States	General population	High
McHugh 2019 [72]	Association of daily co-payments with use of hospital care among Medicare Advantage enrollees	Retrospective database analyses	United States	General population	High
Mehta 2017 [73]	A Dynamic Model of Health Insurance Choices and Health Care Consumption Decisions	Economic model	United States	Chronic disease	High
Reed 2017 [74]	Value-Based Insurance Design Benefit Offsets Reductions In Medication Adherence Associated With Switch To Deductible Plan	Natural experiment	United States	Chronic disease	High
Remmerswaal 2019 [75]	Cost-Sharing Design Matters: A Comparison of the Rebate and Deductible in Health Care	Retrospective database analyses	Netherlands	General population	High
Sabik 2020 [76]	Co-payment policies and breast and cervical cancer screening in Medicaid	Retrospective database analyses	United States	Cancer (breast and cervical)	Medium
Schneeweiss 2007 [77]	Adherence to statin therapy under drug cost sharing in patients with and without acute myocardial infarction: a population-based natural experiment	Natural experiment	Canada	Statin users after myocardial infarction	High
Schneeweiss 2007 [78]	Adherence to beta-blocker therapy under drug cost-sharing in patients with and without acute myocardial infarction	Natural experiment	Canada	Beta blocker users after myocardial infarction	High
Schubert 2014 [79]	Reducing Public Health Insurance Expenditure: A Numerical Analysis for Germany	Economic model	Germany	General population	Medium
Solanki 2000 [51]	The direct and indirect effects of cost-sharing on the use of preventive services	Natural experiment	United States	Preventative services	High
Tang 2014 [56]	Impact of Medicare part D plan features on use of generic drugs	Retrospective database analyses	United States	Chronic disease	Medium
Wang 2010 [52]	Impact of drug cost sharing on service use and adverse clinical outcomes in elderly receiving antidepressants	Natural experiment	Canada	Depression	Medium
Whaley 2017 [80]	The moral hazard effects of consumer responses to targeted cost-sharing	Retrospective database analyses	United States	Colonoscopy services	High

## Data Availability

Data are available upon reasonable request.

## Appendix A

**Table A1:** Detailed pairwise comparisons of cost-exposure mechanisms based on the reviewed literature.

	**Co-pay** (beneficiaries are required to pay a fixed amount per service received)	**Co-insurance** (beneficiaries are required to pay a fixed percentage of costs)	**Deductible** (beneficiaries have to meet a minimum OOP spending threshold before the insurer begins to pay)
**Co-insurance** (beneficiaries are required to pay a fixed percentage of costs)	Those with co-insurance or co-pays generally have the same behavioral pattern. However, those under co-insurance have 10% lower compliance than those under co-pay [50]. Co-pays in group model health management organizations had a smaller impact on utilization rates of preventive services than cost-sharing in preferred provider organization/Indemnity plans [51]. Implementing a co-pay policy led to decreased antidepressant initiation but not increased use of hospitalizations, nursing home admissions, and physician services. Implementing a co-insurance/deductible policy reduced the growth of antidepressant dispensing but did not increase the physician services or adverse events [52]. Co-pay measures had a greater effect on initial medication nonadherence on those who were poor and pensioners compared to other subgroups. When co-insurance policies for pensioners were applied, initial medication nonadherence abruptly increased in the poor population, although coverage for poor pensioners did not change [59]. Monthly inhaler spending was reduced CAD 1.927 million 2003 by the co-pay plan and 6.129 million by the deductible and co-insurance plan [60]. Fixed co-pay plans were associated with a lower risk of treatment discontinuation but higher total mean health care compared with co-insurance plans [64]. Under a 20% co-insurance plan those who took oral hypoglycemic agents or only medical nutrition therapy decreased their self-monitoring blood glucose compared with those who received their test strips for free under a USD 0 co-pay plan. The mean daily test strip use and the prevalence of self-monitoring blood glucose were consistently lower among those paying the higher out-of-pocket co-pay [67]. After the implementation of a generic pricing policy, seniors in provinces with co-insurance have lower out-of-pocket costs than those who faced fixed co-pay [70]. Fixed co-pay policy and a subsequent co-insurance policy reduced adherence to statin therapy by 5 percentage points. For low-income patients, the co-pay policy reduced statin adherence but not the subsequent co-insurance policy [77]. Fixed patient co-pays and co-insurance policies had little negative effect on adherence to beta-blocker therapy or the initiation of beta-blockers after acute MI. The adherence of low-income patients was not impacted by cost-sharing [78]. Co-pays and co-insurance (for plans that cover both in- and out-of-network services) and deductibles (for plans that cover in-network services only) are associated with small decreases in in-network individual psychotherapy visits and associated total expenditures. Findings for co-pays and co-insurance were significant among the high-income/net-worth sample but not among low-income/net-worth sample [62]. Plans with higher deductibles and higher co-pays resulted in a greater reduction in low-value services. A plan with a higher cost-sharing with co-insurance had a greater reduction in low-value services than plans with lower cost-sharing and no co-insurance but not a greater reduction compared to a plan with high cost-sharing and high co-pays [65]. The more comprehensive plan (higher premium, lower co-pay, lower co-insurance, lower deductible) resulted in greater consumption of all types of care visits resulting in higher total costs [73].		
**Deductible** (beneficiaries have to meet a minimum OOP spending threshold before the insurer begins to pay)	Greater prescription cost sharing through deductible resulted in lower utilization of high-value care as defined by drugs used for chronic conditions [53]. Both co-pay and deductible and co-insurance reduced monthly spending on inhalers, but the deductible and co-insurance plan had a larger effect [60]. The change from a deductible at admission to a per diem co-pay (charge per day) was associated with significant declines in inpatient admissions, inpatient days per 100 enrollees, and the probability of hospital admissions, while there was no significant change in length of stay between the plans [72]. With a moderate increase in co-pays rather than the introduction of deductibles, relative equivalent variations are less positive and even negative in some cases. However, in the presence of a health premium, deductibles and raised co-pays raise household welfare for employed individuals [79].	The more comprehensive plan (higher premium, lower co-pay, lower co-insurance, lower deductible) resulted in greater consumption of all types of care visits resulting in higher total costs [73].	
**Tiers** (placing drugs or health services into different degrees of coverage based on criteria determined by the insurer)	As co-pays increase for enrollees in multi-tier plans, their overall use of antihypertensives reduces. Raising co-pays within a single-tier formulary has a relatively modest impact on use of antihypertensives compared with doing so in multi-tier plans, especially those with higher brand/generic differentials [66]. Changing from a single-tier or 2-tier formulary to a 3-tier formulary was associated with a decrease in total drug spending of about 5–15%. Changing to an incentive formulary with higher co-payments was accompanied by an inconsistently small decrease in the use of nonformulary selections and a concomitant increase in both generic and formulary-preferred utilization [69]. The group that had tiered co-pays for outpatient visits but had them waived for preventive care had the highest rates of screening for both Pap smears and mammograms. However, women with co-pays for preventive services are somewhat more likely to receive Pap tests compared with those who face no co-pays for any services [76].	Changing from a 3-tier co-pay to a 4-tier co-insurance benefit design slowed the growth of total per member per month expenditures for all drug classes and the growth of per member per month utilization and expenditures for 3 classes of essential medications but did not have a significant impact on overall per member per month utilization [68]. Relative to plan sponsors with flat 3-tier designs, those with flat 2-tier designs, co-insurance, or tiered co-insurance had generic fill rates that were 2.0 and 1.2 percentage points lower [71]	Tiered plans lead to lower average spending than deductible plans at a lower OOP cost to consumers [58].
**VBID** (making coverage and reimbursement decisions based on perceived value of a good or service, rather than total cost to the insurer)	Applying VBID co-pay structures to pharmaceutical expenditures and health services leads to increased life expectancy gain attributable to health care and varying effects of overall health expenditures when compared to a non-VBID co-pay structure [55].	The implementation of VBID program via additional co-pays for low-value services (sleep studies, advanced imaging services, endoscopies, and surgeries), significantly reduced their use but did not lead to overall cost savings compared to a co-insurance plan [63].	Patients under VBID maintained comparable levels of adherence after switching to a deductible plan compared with statistically significantly greater decreases in adherence in patients under a deductible plan with no VBID. VBID led to improvements in adherence for patients in higher SES neighborhoods but not those in lower SES neighborhoods [74].
**Consumer rebate** (providing money back to beneficiaries who stay below a predefined threshold of health care utilization)	***No papers with this comparison***	***No papers with this comparison***	Cost-sharing decreases health care expenditures under both a no-claim refund and a deductible policy, but the decrease is much larger under a deductible policy [54]. Both the deductible and rebate plans had no impact on pharmaceutical expenditures, there was a stronger effect for the deductible than the rebate for physiotherapy, and for lower-income hospital care, there was a significant and large difference between the deductible and rebate [75].
**Prior authorization** (requiring pre-approval from the insurer for prescriptions to qualify for coverage) and **Step therapy** (requiring use of lower cost therapies for a given condition before “stepping up” to more expensive therapies)	Enrollees in plans with use of prior authorization had significantly higher odds of using generics for antidepressants, antidiabetics, or statins than those in plans without prior authorization requirements. Beneficiaries in plans using step therapy were more likely to use generic antidepressants and generic statins, but these policies were not significantly associated with use of generic antidiabetics [56]. Requiring step therapy or prior authorization increased the use of generic statins compared to real-world cost sharing [57].	***No papers with this comparison***	***No papers with this comparison***
**Reference pricing** (tethering reimbursement rates to specific reference points rather than individual provider charges)	***No papers with this comparison***	The addition of reference pricing to real-world cost sharing increases demand for low-priced providers and leads to a sizable reduction in the average cost per colonoscopy [80].	The addition of reference pricing to real-world cost sharing increases demand for low-priced providers and leads to a sizable reduction in the average cost per colonoscopy [80].
**Narrow Network/ Physician Panel**	***No papers with this comparison***	***No papers with this comparison***	Narrow panel health plans do better at reducing costs and utilization than high cost sharing plans, relative to preferred provider organizations [61].
